# Social capital and the diffusion of learning management systems: a case study

**DOI:** 10.1186/s13731-020-00139-z

**Published:** 2020-11-23

**Authors:** Bill Boland

**Affiliations:** Xi’an Jiaotong-Liverpool University (XJTLU) Entrepreneur College (Taicang), 111 Renai Road, Dushu Lake Science and Education Innovation District, Suzhou, Jiangsu Province, 215123 China

**Keywords:** Diffusion, Adoption, Innovation, Learning management systems, Social capital

## Abstract

The diffusion and adoption process of a learning management system (LMS) at higher education institutes faces several obstacles; some of which are unique to contexts while others are shared experiences. This diffusion case study compares the adoption process of the LMS Blackboard at two universities, Texas A&M University and Monash University in Australia, investigating the factors which impacted the adoption of the innovation at each context as well as the extent to which social capital influenced the diffusion process. The study specifically examined the different adopters involved, the objections raised, the barriers encountered, and the significant factors either resulting in the success or the failure of the innovation, employing a social capital-infused theoretical framework of diffusion within organizations outlined by Frank, Zhao, and Borman (Sociol Educ 77:148–171, 2004). Primary and secondary data were analyzed and examined from three peer-reviewed, empirical articles for comparison within the study.

Findings highlighted strong alignment with Rogers’ (Diffusion of innovations, 2003) diffusion of innovations theory as well as the importance of social capital maintained by Frank and colleagues (Sociol Educ 77:148–171, 2004). Though describing different adoption processes and factors, each context supported the universal idea behind diffusion theory that members of a social system communicate an innovation through social channels over time and that innovators and early adopters play a vital role in this process (Rogers, Diffusion of innovations, 2003). As higher education institutes advance further into the twenty-first century and adopt more innovations within their learning frameworks and systems, this diffusion case study stresses the importance of understanding diffusion theory, having an in-depth knowledge of the stakeholders involved in the adoption process, and creating and implementing a meticulous diffusion plan to ensure a successful diffusion and adoption process.

## Introduction

This study investigates the diffusion of learning management systems (LMSs) in educational institutions through the review, analysis, documentation, and evaluation of the innovation adoption process in the select contexts. Four significant factors impact diffusion and the adoption of an innovation, which include the actual change, the innovation’s communication process, the period for the innovation process, and the social system of the innovation’s integration (Rogers, 2003; Surry, 1997). The focus of this study is to examine the adoption process of LMSs regarding the different adopters (i.e., early, middle, and late) involved, the objections raised, the barriers encountered, and the significant factors either resulting in the success or the failure of the innovation. Specifically, the study seeks to address the following research questions:

RQ1: What factors impacted the adoption of LMSs in the studied contexts?

RQ2: To what extent did social capital influence the adoption of LMSs in the studied contexts?

### What are LMSs, and why are they used?

LMSs are defined as software applications that support the administration, documentation, internal review and tracking, reporting, and delivery of educational and training course-related materials, allowing communication between students and faculty in virtual spaces (Sanga, 2016; Walker & Lindner, 2015; Watson & Watson, 2007). Standard features include an online technical framework and infrastructure that assists with the facilitation of individually paced learning through individual lessons incorporated to an overall program with instructional objectives and a data analytics management system for monitoring, tracking, and reporting different aspects of student learning (Bailey, 1993; Fındık-Coşkunçay, Alkış, Özkan-Yıldırım, 2018; Gilhooly, 2001; Watson & Watson, 2007). There are currently three primary types of LMSs on the market, including (a) cloud-based, (b) open-source, and (c) proprietary (Center for Educational Innovation, n.d.; Dobre, 2015).

These platforms changed the manner in which higher education institutions, as well as businesses, provided educational opportunities to their students and employees, offering easily accessible online options of learning interaction to substitute for face-to-face components. While LMSs have been widely adopted throughout different educational institutions due to their accessibility, cost-effectiveness, and quality, they have encountered challenges and failure, and therefore, the adoption process of LMSs requires further investigation (Fındık-Coşkunçay et al., 2018). While multiple LMS options exist and functions are improving, many LMS platforms remain generic in what options and abilities, such as collaborative tools, schedulers, and discussion forums, they offer to students (Black et al., 2007; Mkhize, Mtsweni, & Buthelezi, 2016). Failures of LMSs to be adopted often related to a lack of technology literacy skills among users (Mkhize et al., 2016). An Educause Center for Analysis and Research 2014 report revealed that 99% of higher education institutions employ LMSs, which 85% of faculty (Brooks, 2015) and 83% of students use (Center for Educational Innovation, n.d.; Dahlstrom, Brooks, & Bischel, 2014; Lang & Pirani, 2014; Rhode et al., 2017).

This study focuses explicitly on variations of the Blackboard LMS, which was created by Michael Chasen and Matthew Pittinsky in 1997. With an initial client base of only 15, the organization experienced rapid growth over the years to around 9300 educational institutions, making it one of the leading companies in its field related to LMSs and LMS-related services around the world and offering a fully integrated, comprehensive information technology service package through its Blackboard Learn service (Blackboard, 2018; Walker and Lindner, 2015). In higher education, Blackboard currently holds the highest market share at 31.9% or 1194 institutions (Center for Educational Innovation, n.d.).

### Theoretical framework

With the increased adoption of online learning approaches through LMSs across the globe in educational as well as other organizations, it is important to investigate the theoretical underpinnings which impact this process. One of the essential perspectives to examine is the different community frameworks and structures and how these social networks may or may not affect this adoption process. In the related LMS diffusion literature, a variety of different theoretical approaches have been applied by researchers to investigate this process. The lens selected for this study emerges from the theory of social capital, which highlights the expertise and resources one can access within their social network and leverage, in one manner or another, to enact change (Coleman, 1988; Lin, 2001; Woolcock, 1998). Specifically, the theoretical framework of this study is grounded in the social capital-infused theoretical model of diffusion within organizations outlined by Frank (2004). Rogers (2003) highlighted that the process of an innovation’s diffusion involved how it was communicated across various channels in an organization over a period of time by the social stakeholders and adopters involved in its adoption process. While Rogers’ diffusion in organizations theory is highly applicable in hierarchal structures, educational institutes are a more complex ecological system of decision-making and influence, requiring the inclusion of social pressures and school-specific aspects influencing the innovation adoption process (Frank et al., 2004). Frank and colleagues highlighted several key elements, including that members of a school obtain benefits from it, can exert social pressure for change, and share the same organizational fate, therefore making them more likely to support others in the adoption process, all of which combine under the theoretical framework of social capital.

When viewing an institution as a whole, staff are linked by their shared connection to the institution’s social system as well as the success or failure of its endeavors (Frank et al., 2004). As such, social pressures within an organization often drive the direction of innovative change from the top down through champions and change agents. Networked interactions of social capital within an institution help one “understand the transitions between the macrolevel social entity of the organization and the micro level action of independent individuals” (Frank et al., 2004, p. 162). It is important to note, however, that social capital is specific to an institution. Therefore, the successful implementation of an innovation in one organization may not necessarily have the same results in another. Additionally, the “flow of social capital is guided by social exchange” (Frank et al., 2004, p. 163). It is linked to the relationships and overall sense of community and collegial environment which exists in an institution. An implication highlighted by Frank et al. (2004) noted that change agents could leverage the readily available resource of social capital as a tool to facilitate the implementation of a particular innovation in an institution, such as the integration and use of an LMS as discussed in this study, promoting macro- and micro-level change.

## Method

This study uses a comparative case study methodology to examine the adoption and diffusion process of LMSs within two contexts, formulating and assessing generalizations that existed across the selected cases. Specifically, it aligns with the innovative comparative case study approach maintained by Bartlett and Vavrus (2017). This approach employs two logics at the same time, including (1) standard compare and contrast with (2) tracing execution across sites and scales. The rationale for this selection is related to its alignment with ideas of diffusion with a social, networked system. The case study comparison approach suggested by Bartlett and Vavrus (2017) allows for “simultaneous and overlapping attention to three axes of comparison” (p. 15). First, a horizontal perspective reviews how the development of similar policies or phenomena (e.g., the introduction of an innovation) occur in locations that share a connection (e.g., higher education institutions engaged in online learning) and are socially produced (e.g., diffusion through the theoretical perspective of social capital). Second, a vertical analysis focuses on tracing the process across scales and levels within an institution. And, finally, a transversal perspective examines the phenomena or diffusion process across time. Each of the three areas in this analytical approach aligns strongly with the purpose of this study. The following section details the methods employed for the study’s analysis.

### Case selection

The researcher sought to identify higher education context case studies that implemented variations of Blackboard as an LMS. Several factors were considered when searching for and selecting the cases. First, the researcher limited the selection to higher education institutions with related case studies conducted within the last 15 years. Institutions from any global location were considered viable. Second, the case studies in the institutions needed to be of significant size and longevity to provide data related to adoption practices to align with the comparative case study approach. Third, it was important for the case studies to focus on similar LMSs to offer an accurate frame of reference regarding the comparison of adoption factors. A final practical factor involved access to enough primary and secondary data to provide a comprehensive analysis. After a search through the Education Resources Information Center (ERIC), the research identified two candidates with related case studies on which to base the investigation: Texas A&M University and Monash University.

The first context examined involved a comparison of the adoption of the current Blackboard Vista LMS, which was an end-of-life product, at Texas A&M University in the USA by 57,230 users consisting of faculty (*n* = 3949), staff (*n* = 5997), and students (*n* = 47,230) during the spring 2011 semester and its potential replacements Moodle, Sakai, and Blackboard Learn. The primary data source is a peer-reviewed, empirical study by Walker and Lindner (2015), which employed a stratified census approach collecting data using open discussion forums, a Qualtrics survey, and data from the university student information system database. A total of 579 faculty, 1180 staff, and 4173 students responded to the survey. Secondary sources (Blackboard, 2016; Santos, 2017) include website articles connected to the university as well as Blackboard. There was not a lot of information available on the adoption process of the Blackboard LMS in this context. While the researcher discovered an evaluation report as a resource in the Walker and Lindner study, it is no longer available through the university.

The second context explored involved a study of the adoption process of WebCT Vista, also known as Blackboard Vista, at Monash University, an Australian multi-campus institution with over 2500 teaching faculty and 53,000 students from more than 100 countries. Two peer-reviewed, empirical studies provide the primary data. Benson and Palaskas (2006) investigated the piloting and evaluation phase of Blackboard Vista at Monash University through a mixed methods approach, exploring the adoption and diffusion of technological change across two-semester phases. The first phase involved 1600 students across four campuses in 2004 and focused on staff viewpoints. The second involved 5500 across all schools, including those in Malaysia and South Africa, and highlighted both staff and student perspectives. Data collection strategies included project documentation analysis, interviews, surveys, and focus groups. The second study by Samarawickrema and Stacey (2007) employed a case study approach using Rogers’ (2003) theory of diffusion as a lens of investigation to reveal factors influencing the adoption of Blackboard Vista by faculty. The 22 instructors described as innovative were purposively selected from the six Australian campuses that participated. Data collection occurred through interviews, the examination of artifacts, and field notes. Secondary sources for this study (Bertsche, 2017; Beyersdorf, 2017) are website articles connected to the university. There was not a lot of information available on the adoption process of the Blackboard Vista LMS in this context.

### Data collection and coding

Primary and secondary data were analyzed from three peer-reviewed, empirical articles for comparison, searching for related themes. The researcher employed a comparative framework which highlighted four key thematic areas which aligned with Rogers (2003) adoption process, including (1) the motivation to adopt, (2) the decision to adopt, (3) the adopter categories, and (4) the implications of the adoption process as noted in Table 1.

**Table 1:** Thematic categories

Category	Description
Motivation to adopt	Motivation to adopt involved a review of why the contexts were influenced to adopt the particular LMSs in question, focusing on a variety of factors. Five attributes of an innovation that effect adoption that were reviewed related to Rogers’ (2003) diffusion of innovations theory included (1) relative advantage, (2) compatibility, (3) complexity, (4) trialability, and (5) observability.
Decision to adopt	Decision to adopt was an analysis reflecting on where the decision to employ the selected LMSs emerged within the contexts and how that decision was diffused through the contextual social environment.
Adopter categories	Adopter categories involved an examination related to Rogers’ (2003) adopter categories associated with the diffusion of an innovation, including (1) innovators, (2) early adopters, (3) early majority, (4) late majority, and (5) laggards.
Implications of the adoption process	Implications of the adoption process related to what discernible outcomes the adoption of the LMSs had on the contexts.

First, the researcher thematically coded the case studies using the four primary a priori categories (i.e., motivation to adopt, decision to adopt, adopter categories, and implications of the adoption process) as well as inductive coding related to grounded theory to identify emerging categories and subcategories to add to the code system (Glaser & Strauss, 1967). The researcher read and reread the studies a total of five times to identify the emergent categories. Then, the researcher sorted statements and data into these emergent categories. Next, the researcher noted the common themes within the categories and further sorted and differentiated them into subcategories. The analysis necessitated constant comparison to consolidate similar concepts into overarching ones, allowing for theme differentiation (Corbin & Strauss, 2008). Through the thematic comparison of the adoption process in these two contexts in the related case studies, this study addressed the two key research questions related to factors that impacted adoption as well as the influence of social capital in different settings, highlighting how and why each of the particular processes worked or failed to work.

## Result and discussion

In the following section, the results of the adoption process in both contexts are compared and contrasted related to the motivation to adopt, the decision to adopt, the adopter categories, and the implications of the adoption process.

### Motivation to adopt

Within Texas A&M, the innovation characteristics that influenced the adoption of the Blackboard Vista LMS included relative advantage and compatibility (Walker & Lindner, 2015). Aligning with the current practices at the university, the LMS allowed faculty to deliver instructional content better and engage students in collaborative discussions. For students, it offered the improved ability to learn outside of the classroom setting as well as provided new research and learning channels. An ease of use analysis of the LMS highlighted that all participants ranked the LMS elements as either very easy or easy to use, eliminating complexity as a potential barrier to adoption. Participants, however, noted that new LMS features, functions, and tools were not necessarily a factor in adoption.

In the Monash University context, Benson and Palaskas (2006) detailed that the administration of the university set up the small, structured pilot program, so the motivation to adopt by participants was decided by top-down decision, allowing them little individual input in the decision process. Within the pilot, however, complexity and compatibility played an essential role with participants only engaging in basic features that aligned with their current needs and practices and highlighting the need for future training and professional development for more significant pedagogical engagement with the LMS (Benson & Palaska, 2006).

Similar to Benson and Palaska (2006), Samarawickrema and Stacey (2007) studied the motivation to adopt in Monash University from a case study perspective of innovation educators, highlighting the influence of five important attributes and participants’ perspectives on their impact: (a) relative advantage (*n* = 10), (b) compatibility with existing values (*n* = 7), (c) ease of use (*n* = 3), (d) trialability (*n* = 4), and (e) observability (*n* = 4). Additional key reasons affecting the motivation to adopt included top-down authority innovation directions, demand by students at the university, economic and political necessity, communication opportunities, and supportive social systems (Samarawickrema & Stacey, 2007). Five major categories of reasons for adoption emerged, including institutional reasons, technology reasons, the influence of colleagues/network, the influence of research literature, and personal reasons, with institutional reasons as the dominant factor (Samarawickrema & Stacey, 2007). In a conference presentation, the importance of leading by example was highlighted as a key influential motivational factor in the Monash University Arts Educational Design team regarding LMS and e-learning adoption, describing faculty as more likely to embrace cultural change if it is manageable, supported by the university, and endorsed by colleagues (Beyersdorf, 2017). Without contextual and collegial support, the adoption process could run into a fundamental barrier among potential stakeholders as the organizational culture would seem aligned against the change. In particular, the influence of colleagues/network as a reason for adoption coupled with the importance of organizational culture regarding embracing an innovative change in the case of Monash University reinforced ideas of social capital-infused model highlighted by Frank et al. (2004). Social networks and exchange represent critical elements to drive innovation adoption from a macro to a micro organizational level.

### Decision to adopt

At Texas A&M, the Blackboard Vista LMS was first employed in 1998 as a top-down, authority-innovation decision associated with a teaching with technology initiative implemented to improve the teaching and learning process for faculty and students (Walker & Lindner, 2015). Staff and students, however, were allowed the freedom to choose whether or not to utilize the system once it was officially implemented, removing a potential barrier of resistance to a top-down directive. Dr. James Snell, the Distance Education Department’s current director, described a lack of acceptance on the campus at first in 2002 to the new centralized educational technology resource, but now, the department has been successful in fostering significant support for online education among faculty and students (Santos, 2017). Usage by faculty and students improved each year since 2002, engaging 92% of the study’s participants in regular use by 2011 (Walker & Lindner, 2015). In a report by Blackboard in 2016, Dr. Lauren Cifuentes, Director of Distance Education and Learning Technologies for Texas A&M University—Corpus Christi, described the Blackboard environment as one in which the faculty and students “live in” (Blackboard, 2016, p. 1). Despite starting as a top-down, authority-innovation decision, the Blackboard system became integral for faculty and students engaged in education work at the university over the years (Blackboard, 2016).

Similarly, in the Monash University context, Benson and Palaskas (2006) described a top-down, authority-innovation decision as the administration employed two pilot programs of the Blackboard Vista LMS with select faculty and students in the two semesters of 2004 to evaluate the program. The pilot program involved mandatory participation of 15 units in the first semester, followed by 80 units in the second with the objective of informing full implementation as connected to the selected units. Samarawickrema and Stacey (2007) also maintained that institutional reasons, specifically top-down, authority-innovation directives, were the major influential factor in the decision for faculty to adopt.

### Adopter categories

Walker and Lindner (2015) did not explicitly identify the involvement of the adopter categories at Texas A&M University. The authors, however, did note the critical importance of innovators and early adopters, users classified as possessing 2 to 4 years of experience with an LMS, as essential to the piloting process and initial adoption period. These individuals played a fundamental role in establishing the foundation of the LMS at the university and expanding its use through the identification of early problems as well as outreach and social connections to colleagues, connecting to ideas proposed by Frank et al. (2004) regarding a social capital infused theoretical model of diffusion within organizations. Though other adopter categories were not mentioned, one can infer by the spread of the adoption to 92% of faculty, staff, and students participating in Walker and Lindner’s study that the initial work by the innovators and early adopters was successful in reaching both early and late majority adopters through social influence and the collegial exchange of ideas. The small remaining percentage of users who did not adopt the use of the LMS reflects the adoption bell curve described by Rogers (2003), pinpointing this group as laggards behind the overall majority.

Whereas sources on the Texas A&M context remained somewhat vague on adopter categories, Benson and Palaskas (2006) detailed Monash University’s engagement of innovators as they piloted the new Blackboard Vista LMS in their context. This group, as described by Rogers (2003), involved those interested in testing new technology innovations. Considering the units were selected by the university at random, it is indeterminate, however, how widespread innovators were within the pilot program and which other adopter groups were present. While Samarawickrema and Stacey (2007) did not explicitly detail any adopter categories either, the study’s description of the case study involving innovative academics suggests the participants would fall mainly into the category of innovators with some potentially as early adopters in the context. Beyersdorf (2017) also highlighted the critical importance of those who lead by example (e.g., faculty innovators and early adopters) to the adoption process through social capital and interaction at Monash University, making the cultural shift easier for peers.

The success of the adoption process at Monash University was reflected in 2017 when Monash College, a pre-university feeder institution for the university, was awarded the 2017 Blackboard Catalyst Award, highlighting an institution which fully integrated the LMS delivery model as well as supported and enhanced faculty and staff with high-quality professional development (Bertsche, 2017). The award highlights the broad adoption of the program in all areas of Monash University over the years, suggesting outreach to all categories of adopters (i.e., innovators, early adopters, early majority, and late majority) as well as intense training programs for remaining laggards. While Texas A&M University’s adopter categories were harder to track, resources related to Monash University highlighted the vital importance of innovators and early adopters in the innovation’s initial stages and the long-term expansion impact they had with other groups.

### Implications of the adoption process

From the Texas A&M University context, the long-term engagement with the Blackboard LMS, particularly the ease of use analysis, described a high level of self-efficacy amongst faculty and student users and therefore a potential easy ability to adopt a new LMS, such as Moodle, Sakai, or Blackboard Learn (Walker & Lindner, 2015). Even though new features of an LMS did not influence adoption, administration leaders and program vendors need to highlight the relative advantage of new necessary elements on an LMS to ensure a connection to users and improve the adoption rate (Walker & Lindner, 2015). The absence of definite relative advantages and benefits related to the adoption of innovation can frequently lead to rejection by potential users, hindering the diffusion process (Rogers, 2003). Walker and Lindner (2015) maintained the vital importance of higher education institutions to collaborate with innovators and early adopters to identify relative advantages of a new LMS and accelerate the adoption process to other users by working as change agents, which aligned with the results of Benson and Palaskas (2006). The essential nature of change agents and their ability to engage in a shared network to facilitate effective change highlights the importance of social capital and influence in the adoption process noted by Frank et al. (2004). Context politics, including top-down authority adoption directives, funding, and internal faculty relationships and pressure, were also pointed out as significant adoption factors in Monash University by Samarawickrema and Stacey (2007), aligning with the social capital ideas of Frank and colleagues. Faculty relationships and social pressure represented critical areas of shared exchange impacting the adoption process of the LMS, specifically related to the macrolevel social environment and community of the institution and the microlevel actions of individual faculty and staff. When top-down authority adoption directives and internal faculty relationships and pressure are considered together, they pinpoint a potential combined institutional strategy, which incorporates both the organizational level as well as micro to macro organic growth led by influential staff members. This type of plan could maximize social capital as a vital resource along the lines described by Frank et al. (2004).

Walker and Lindner (2015) also described the importance of system usage related to different LMS features as high utilization can be attributed to relative advantage, and therefore, those favors will work in support of the adoption process. A lack of inclusion of these high-value features would result in an important barrier to the innovation’s diffusion. Walker and Lindner pinpointed innovators and early adopters as the innovation champions who enhance credibility and persuasion among other users through social exchange and engagement by helping showcase relative advantage, an absence of complexity, and observability, all key elements of change described by Rogers (2013). Their ability to showcase these different key aspects within their social network explains how the transition from micro to macro adoption increases within an institution as they influence their colleagues in the manner detailed by Frank et al. (2004). While training is essential to the learning process, a training requirement or overly complicated training for the LMS could also work as a barrier for adoption, decreasing ease of use of the system (Benson & Palaskas, 2006; Walker & Lindner, 2015). Through the lens of actor-network theory, Samarawickrema and Stacey (2007) described that faculty respondents unanimously noted that the following factors influenced their ability to use LMS approaches: (a) time constraints, (b) workloads, (c) the reconfiguration of learning materials for the LMS, (d) the demand for research output, (e) professional development, (f) the need for new skills, (g) professional exposure, (h) intellectual property issues, (i) institutional policy issues, (j) funding, and (k) staff attitudes. The administration and leadership of higher education institutions can foster an environment conducive to innovation adoption through the comprehensive consideration of these institutional factors as well as top-down organizational planning to effectively cascade the innovation down through the institution’s networks, channels, and stakeholders (Samarawickrema & Stacey, 2007). A strong, collaborative relationship between adoption teams and academic staff is essential for the diffusion of technology-related innovations, such as an LMS, as these fundamental staff act as conduits for sharing and promoting the change to other faculty (Beyersdorf, 2017), associating with the importance Frank et al. (2004) placed on social capital in the adoption and diffusion process.

### Adoption process of LMSs

Several studies have described the adoption process of various LMSs within different contexts (Arpaci, 2017; Benson & Palaskas, 2006; Chan & Ngai, 2012; Fındık-Coşkunçay et al., 2018; Lwoga, 2014; Mkhize et al., 2016; Rhode et al., 2017; Rucker & Downey, 2016; Samarawickrema & Stacey, 2007; Walker & Lindner, 2015), identifying a variety of reasons connected to Rogers’ (2003) diffusion of innovation theory that adopters embrace an innovation. This study has focused on the Texas A&M University and Monash University contexts, two higher education institutions using a variation of the Blackboard LMS educational technology innovation with faculty and students. As Rogers describes, adopters fall into five different categories based on when they use and implement an innovation, including (a) innovators, (b) early adopters, (c) early majority, (d) late majority, and (e) laggards. Walker and Lindner (2015) highlighted the critical importance of innovators and early adopters in the adoption of the Blackboard LMS at Texas A&M University, and Benson and Palaskas (2006), Samarawickrema and Stacey (2007), and Beyersdorf (2017) noted the importance of innovators in the adoption of the Blackboard Vista LMS at Monash University. The adoption process aligns with an s-shaped, standard bell curve, highlighting the general flow of adoption as more and more individuals in a social system embrace the use of an innovation (Rogers, 2003). Data collected on Monash University reflected this bell curve, social adoption process with the university winning awards for its widespread integration and use of Blackboard (Bertsche, 2017). Rogers also detailed innovation adoption as involving five general stages, including knowledge, persuasion, decision, implementation, and confirmation, covering the entire process from initial awareness to full usage. The early development of the knowledge, persuasion, and decision stages were evident in data collected on Texas A&M (Walker & Lindner, 2015) as well as the confirmation stage (Blackboard, 2016; Santos, 2017), while all of the five general stages were reflected to some degree in adoption data from Monash University (Benson & Palaskas, 2006; Bertsche, 2017; Beyersdorf, 2017; Samarawickrema & Stacey, 2007).

Antecedents to the adoption of new information technology system practices, such as LMSs, include, but are not limited to, perceived advantages and user characteristics (Arpaci, 2017; Chan & Ngai, 2012; Fındık-Coşkunçay et al., 2018; Lwoga, 2014; Monett & Elkina, 2015; Rogers, 2003; Rucker & Downey, 2016; Walker & Linder, 2015), social pressure (Benson & Palaskas, 2006; Flanagin, 2000; Frank et al., 2004; Samarawickrema & Stacey, 2007; Walker & Linder, 2015), and organizational and individual readiness (Chan & Ngai, 2012; Liu, 2005; Samarawickrema & Stacey, 2007; Walker and Lindner, 2015). Perceived advantages, or relative advantages as referred to by Rogers (2013), were described in both Texas A&M University and Monash University as key to the adoption process (Benson & Palaskas, 2006; Samarawickrema & Stacey, 2007; Walker & Linder, 2015). At Texas A&M University, relative advantage translated for faculty as an improved manner to deliver instruction and promote student engagement, while the LMS provided a more accessible and flexible learning environment for students (Walker & Lindner, 2015). At Monash University, relative advantage by the Blackboard Vista LMS for faculty related to its beneficial alignment and compatibility with current institutional practices, providing better communication channels and course delivery and outreach (Samarawickrema & Stacey, 2007).

Each context also highlighted the importance of social pressure, technical compatibility, and management as having significantly more influence on the adoption by early adopters than late adopters (Benson & Palaskas, 2006; Samarawickrema & Stacey, 2007; Walker & Linder, 2015). The authors all noted the impact of institutional and social pressure in the higher education adoption process, maintaining that influential, champion educators were crucial in the early adoption process to disseminate the spread of innovations amongst other faculty and students. This idea aligns with the social capital-infused theoretical model of diffusion within organizations proposed by Frank et al. (2004), pinpointing change agents as critical for the effective exchange of ideas through an organizational social network and community as their influence can effectively and efficiently facilitate the diffusion process. Social exchange through a network by influential faculty can prove critical for the rapid adoption of an LMS as the collegial sharing of ideas can quickly spread, both from a top-down approach as well as from individual to individual. As students and teachers are the primary users of LMSs, they play a critical role in the adoption of any related system (Fındık-Coşkunçay et al., 2018; Samarawickrema & Stacey, 2007; Walker & Linder, 2015).

In a quantitative study focused on the critical success factors for web-based learning management systems, Lwoga (2014) maintained that instructor and system quality factors were significant predictors of perceived usefulness and user satisfaction, which both in turn detailed continual usage by students. Additionally, learner and faculty needs and expectations related to an LMS are of fundamental importance when examining adoption with learner analytics providing a roadmap analyzing behavior and usage needs (Monett & Elkina, 2015; Walker & Linder, 2015). Higher education institutions must consider these user concerns and needs when designing and selecting the proper LMS as well as providing training support for increased usage (Benson & Palaskas, 2006; Samarawickrema & Stacey, 2007; Walker & Linder, 2015). When stakeholders are integrated and unified within the planning and execution stages of a new program strategy, such as introducing an innovation, it leads to more effective results and user buy-in to the process (Bryson; 2004; Bryson & Patton, 2010). Additionally, training support provides critical how-to knowledge regarding information necessary to use the innovation properly, which Rogers (2003) identifies as the most significant form of knowledge required to implement educational technology innovations. However, not all studies of LMS adoption have determined a correlation between positive attitude toward LMS, perceived ease of LMS use, and perceived usefulness and actual use of LMS (Wichadee, 2015), highlighting a need for further investigation.

Rucker and Downey (2016) pinpointed system affordances and complexity of crucial importance when investigating faculty adoption of an LMS, associating with Rogers (2003) that complexity is a vital consideration related to the adoption process of an innovation. If not adequately addressed, an overly complicated system will represent a fundamental barrier in the adoption process, resulting in a lack of engagement by potential users (Rogers, 2003). Walker and Linder (2015) maintained a similar conclusion, pinpointing ease of use as an essential adoption consideration. Complexity and resistance also emerged as critical adoption factors of a mobile LMS with non-users in a higher education Korean study despite the recognition of advantages of the innovative learning approach (Han & Han, 2014). Han and Han (2014) highlighted the essential importance of the perceived ease of use of users related to adoption at all stages, aligning with results noted in the peer-reviewed studies investigating both university contexts (Benson & Palaskas, 2006; Samarawickrema & Stacey, 2007; Walker & Linder, 2015). In a South African study, Mkhize et al. (2016) concluded that the critical deciders of the success of technology innovations are the users who are adopting them, aligning with essential ideas from the course and notably Rogers (2003) related to the diffusion of innovations and the key components impacting adoption, such as compatibility, relative advantage, and complexity. All of these vital components played important roles in the adoption process of the Blackboard LMS at Texas A&M University (Walker & Linder, 2015) as well as Monash University (Benson & Palaskas, 2006; Samarawickrema & Stacey, 2007). A pivotal conclusion highlighted the significant importance of the compatibility of an innovation with the traditional learning values of users, emphasizing the importance of coherence with the existing culture and social systems in the diffusion process (Liao, 2005; Mkhize et al., 2016; Rogers, 2003). The social network in a society and its culture, in this case, higher education institutions, have a significant impact on the ability of an innovation to be adopted for extensive use (Ashley, 2009; Rogers, 2003). Alignment with current practices and needs can be a fundamental determining factor whether or not the innovation will be adopted or not (Rogers, 2003; Samarawickrema & Stacey, 2007; Walker & Linder, 2015).

It is critical to understand the factors that influence the adoption and usage of LMSs. Through this understanding of the adoption process, administrators and LMS providers can offer more beneficial design and implementation aspects as well as more ongoing professional development support for instructors and learners (Rhode et al., 2017; Samarawickrema & Stacey, 2007; Walker & Linder, 2015). These performance improvement aspects will lead to more efficient diffusion of the LMS system in different educational institutions in the future, which will then result in a ripple improvement effect related to the outreach of the entire educational process to students.

## Conclusion

The two contexts examined in this diffusion study showcased a substantial alignment with ideas related to Rogers’ (2003) diffusion of innovations theory as well as the importance of social capital maintained by Frank et al. (2004). While the adoption process of the Blackboard LMS in the contexts was somewhat different due to internal diversity amongst factors, each process highlights common ideas connected to diffusion noted by Rogers. Each setting showcased the notion that the spread of change is a process by which members of a social system communicate an innovation through social channels over time (Frank et al., 2004). While top-down, authority-innovation directives drove the initial adoption processes in the contexts, a review of results showcased that the engagement of important innovators and early adopters, viewed as champions and change agents within their networks, was essential to the diffusion of the innovation throughout the higher education institution. These key adopters also provided critical formative feedback during the evaluation process, allowing the settings to improve the delivery of the innovation to faculty and students and therefore ensure a more efficient adoption process aligned with their concerns and needs.

Despite the different social and cultural backgrounds, the contexts and related resources point toward the universality of diffusion and the importance of understanding the adoption of innovations within higher education to improve the process for all stakeholders significantly.

It is critical to engage key champions, opinion leaders, and early adopters in the adoption process to ensure a smooth and efficient diffusion procedure throughout higher education institutes while using various strategies to mitigate the potential pitfalls. Through a meticulously executed diffusion plan, a university can ensure a successful innovation adoption process and the future goals of the institution toward development and improvement.

The case studies examined support a social capital infused theoretical model of diffusion within higher education institutions while also highlighting other key adoption factors to consider when facilitating an effective adoption process. From the standpoint of contribution to the field, this comparative study has several important implications. First, although the study by Frank et al. (2004) pinpointed the importance of social capital regarding the diffusion of computer technology in K-12 schools, this study highlights how their findings are also applicable to higher education institutions, specifically related to LMSs. This key implication is particularly essential as higher education institutions currently find themselves at a tipping point regarding the implementation of LMSs to support online learning with their students as a result of COVID-19. Second, as social capital within any institution is a cost-free, readily available resource accessible in the form of its existing faculty, it represents foundational element institutions that should consider in organizational plans regarding the implementation of an innovation. Third, with the engagement of the right champions, change agents, and early adopters, an institution can execute a successful adoption process and potentially accelerate it past traditional barriers. As Frank et al. (2004) highlighted, these change agents within the social fabric and network of the institution will be essential for the exchange of collegial ideas. They will help drive the diffusion process through best practices and sharing as well as modeling the successful use of the innovation. Fourth, with online learning on the rise globally and LMSs critical to the delivery of this medium of education, this comparative case study offers vital insight into a variety of essential factors requiring consideration regarding LMS adoption in higher education institutions. Although specific variables will vary from institution to institution, Rogers’ (2003) diffusion of innovations theory, as well as a social capital infused theoretical model of diffusion maintained by Frank et al. (2004), describes a successful pathway for LMS adoption which will face the least resistance while also effectively facilitating the process, providing a roadmap for strategic consideration for global institutions.

## Data Availability

All data generated or analyzed during this study are included in this published article as well as based upon publicly available data in the three referenced, peer-reviewed studies (Benson & Palaskas, 2006; Samarawickrema & Stacey, 2007; Walker & Lindner, 2015).

## Abbreviations

LMS: Learning management system
LMSs: Learning management systems
